# Energy-efficient thermally smart windows with tunable properties across the near- and mid-infrared ranges

**DOI:** 10.1515/nanoph-2025-0219

**Published:** 2025-06-17

**Authors:** Julien Legendre, Georgia T. Papadakis

**Affiliations:** 172281 ICFO-Institut de Ciencies Fotoniques, The Barcelona Institute of Science and Technology, Castelldefels, Barcelona 08860, Spain

**Keywords:** thermal management, smart windows, radiative cooling, phase-change materials, cholesteric liquid crystals

## Abstract

Space heating and cooling account for approximately 15 % of the world’s energy consumption, underscoring the pressing need for improved thermal management. Macroscopic temperature regulation can be significantly optimized by improving radiative heat control, in particular through radiative cooling in summer and sunlight capture for heating in winter. These processes are typically tailored independently and thereby remain passive. In this article, we propose thermally smart windows with radiative properties that adapt to a building’s heating and cooling demands in a tunable manner. To achieve this, one ought to control, simultaneously, the window’s response to near- and mid-infrared (IR) radiation for solar heating and radiative cooling, respectively. We propose device architectures to realize such operations using phase-change materials and liquid crystals. Compared to conventional silica glass, the proposed architectures may reduce the energy demand of buildings at the latitude of Barcelona by more than 40 %, showcasing the potential of tunable materials for radiative thermal management in the energy transition. We discuss that significant promise lies in the development of materials that can warrant near-unity modulation of NIR reflectance, which should be the key property to reach as much as 64 % reduction in energy consumption.

## 1 Introduction

**Figure 1:**
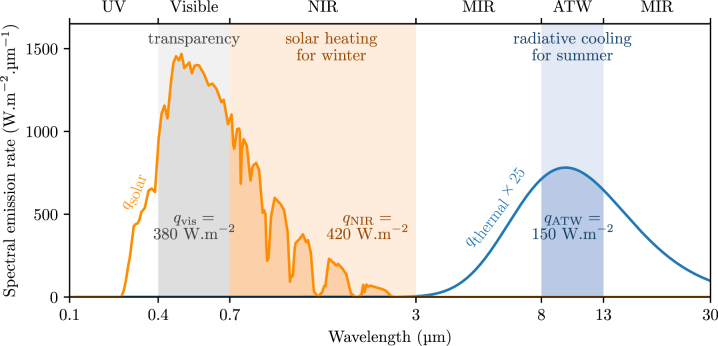
Spectrum of solar radiation upon attenuation by the atmosphere (orange) and of thermal radiation emitted by a blackbody at 300 K (blue). The heat flux values shown in the figure correspond to the integrated power within the indicated spectral ranges. Values referring to the visible and NIR spectral ranges were obtained using Barcelona meteorological data from 1 July 2023 at midday.

Approximately 15 % of the energy consumed worldwide is used for space heating and cooling [1], [2]. This challenge is exacerbated by the greenhouse effect, where heat in the form of thermal radiation is trapped within the atmosphere resulting in increasing cooling needs globally. In an effort to reduce energy demands, radiative heat control holds promise for efficient temperature regulation. Specifically, it is possible to tailor the in-flux and out-flux of thermal radiation to enable passive heating and cooling, respectively. Figure 1 demonstrates the spectrum of solar radiation (5780 K) and that of a blackbody at 300 K. As shown, solar radiation peaks at visible and near-infrared (NIR) frequencies, carrying roughly 800 W/m^2^ of available power density for heating in these two ranges, e.g., for space [3] or water heating [4]. Conversely, making use of the atmospheric transparency window (ATW) in the wavelength range 8–13 µm, thermal radiation from macroscopic object at terrestrial temperatures can be rejected into the cold universe (3 K), enabling radiative cooling [5]. The spectrum of blackbody radiation at 300 K peaks at mid-infrared (MIR) frequencies, near 10 µm (Figure 1), and roughly 150 W/m^2^ of heat can be extracted within the ATW by ensuring high emissivity within this spectral range. With additional high reflectivity of solar irradiance at visible and NIR frequencies, macroscopic bodies can passively reduce their temperature to even below-ambient temperatures [5], [6], [7], [8], [9], [10], [11], [12]. Radiative cooling has been employed for temperature regulation in buildings, by placing such coolers on rooftops [6], which maximizes radiative heat exchange with the sky. Recently, radiative cooling has also been considered for façades [13], [14].

Windows play a crucial role in the temperature regulation of buildings. A standard glass window, composed of silica, operates favorably for solar heating in winter rather than cooling in summer. This is because glasses, other than being transparent at visible frequencies, are also transparent at NIR frequencies. This enables the in-flux of NIR solar photons that account for nearly half of the total solar irradiance (Figure 1). By contrast, conventional glasses do not emit sufficiently within the ATW, making them suboptimal for radiative cooling purposes. As a result, the cooling needs of a building equipped with standard glass windows in an urban environment during summer account for more than double those for heating in winter (see Section 2), in the location of Barcelona. To improve the cooling properties of windows, materials with strong NIR reflectivity and ATW emissivity have been considered [15]; however, such windows present significantly compromised performance during winter. In fact, the deficit of heat required to be supplied in winter can be even greater than the energy saved in summer, in comparison to a standard glazing.

Here, to circumvent the challenge of compromised performance in either winter or summer, we consider actively tunable radiative properties in *smart* windows. Conventionally, the term “smart window” refers to windows that can change their response at visible frequencies from transparent to opaque [16], [17], [18]. Nonetheless, smart windows with actively tunable properties in other spectral ranges have been considered recently for radiative thermal regulation, operating based on an applied voltage, light- or temperature-activation [19], [20]. Smart windows that are transparent to visible light and actively tunable at NIR frequencies [21], [22], [23], [24], [25] or within the ATW [26], [27], [28] have been reported. However, the comparative merits of NIR and ATW modulation for thermal management have never been detailed, and no window possessing tunable properties in *both* of those two ranges has previously been introduced.

In this work, we analyze the potential of simultaneous NIR- and MIR-tunable thermally smart windows for thermal management applications from theoretical and practical perspectives. Considering a building’s annual energy demand for heating and cooling in an urban environment, we quantify the energy gain in terms of consumed electricity using actively tunable windows across the NIR and MIR ranges, as compared to standard glass windows, demonstrating a performance improvement that can reach 64 % for ideal materials. We also identify realistic photonic structures with tunable thermal radiative properties in both NIR and MIR ranges, considering two classes of tunable materials (phase-change materials and cholesteric liquid crystals) and quantifying their potential benefit in energy savings of buildings. We highlight the respective merits and constraints in the mechanisms available for achieving NIR and MIR modulation for temperature regulation and discuss the fundamental difference in maturity and in the materials available for tunable optical response across these two spectral ranges. We conclude that radiative temperature regulation will benefit significantly from the development of materials that can achieve broader NIR modulation of their properties, while available phase-change materials already suffice for implementing MIR emissivity control strategies.

## 2 Heat load estimation

**Figure 2:**
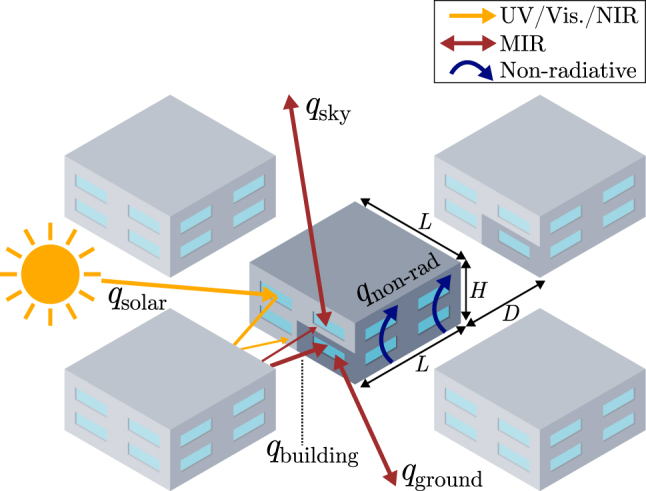
Schematic of the urban environment considered for the calculation of the yearly heat load on a building. The arrows represent the various channels of heat transfer with the building.

First, we study how the radiative properties of a window impact the energy demands of a building for heating and cooling. We consider the building to be located in an urban environment in Barcelona, Spain. To simulate an urban environment, the building is considered to be surrounded by identical copies of itself, with each façade being equally covered by windows and walls, as illustrated in Figure 2. The temperature of the room inside the building is maintained at 20 °C in winter and at 25 °C in summer by means of heating and cooling systems. The heat load of the building is expressed as:

(1) $$q_{\text{load}}=q_{\text{sun}}+q_{\text{sky}}+q_{\text{ground}}+q_{\text{building}}+q_{\text{nonrad}},$$

where the first four terms pertain to radiative contributions, namely heat exchange with the sun, the sky, the ground, and the opposite building, respectively. The last term is related to the conductive and convective heat transfer between the room and the outside air through the façade. The considered solar illumination onto the different façades varies during the day as modeled with the Python package *PVLIB* [29] (see, for example, Figure 1 for the solar spectrum provided by *PVLIB*). The atmosphere is considered to have a unitary emissivity for all wavelengths outside the ATW. Within the ATW, its emissivity is considered equal to 0.2. The ground is simulated by a blackbody with temperature equal to the air temperature, whose hourly variation is taken into account and modeled with the *Meteostat* package [30]. Although air renewal within the building can potentially affect heat regulation, it does not depend on the properties of the window, for which we omit its contribution. Further details on the models considered in each term of Eq. (1) and on the atmospheric properties can be found in Section I of Supplementary Material. In this section, we also outline the influence of cloud coverage on the terms of Eq. (1) and demonstrate that its impact is negligible in the case of a building in Barcelona. Finally, we note that the case of a rural environment is investigated in Section II of Supplementary Material, complementary to the urban scenario discussed here.

**Figure 3:**
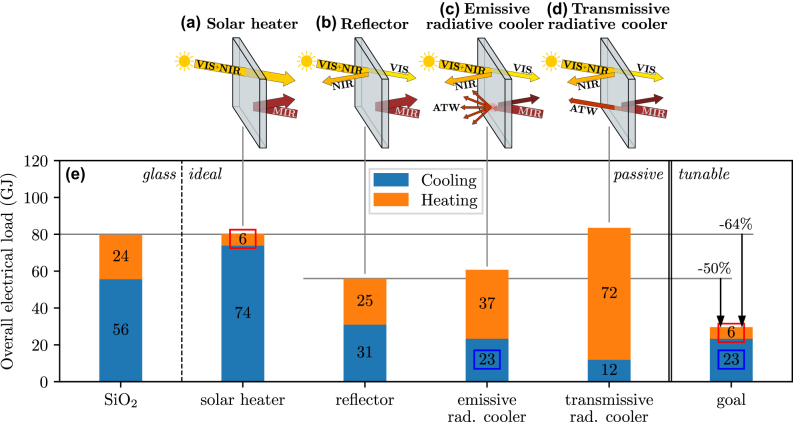
Influence of the radiative properties of windows on the heating and cooling demand of a building. (a–d) Illustration of the radiative properties of the four different kind of static windows with ideal properties. (e) Yearly electrical load of a building due to heating and cooling, considering a building with dimensions *L* = 20 m and *H* = 10 m in an urban environment (5 m distance between buildings). On the right is indicated the load on a building equipped with switchable windows, operating as solar heating windows in winter and as emissive radiative cooling windows in summer.

We consider four different types of ideal static windows, as illustrated in Figure 3. These are (a) a *solar heating* window, which maximizes the transmitted solar radiation in the NIR while reflecting MIR radiation to isolate the room from the exterior environment; (b) a *reflective* window (also called low-emissivity or low-e window), which reflects in the whole IR, thus thermally isolating the room as much as possible; (c) an *emissive radiative cooling* window, which reflects in the NIR while emitting within the ATW to cool the room; and (d) a *transmissive radiative cooling* window, which is similar to (c) but is transmissive rather than emissive within the ATW. All cases of windows ought to remain transparent at visible wavelengths. This is not a common constraint for smart windows, which often switch from transparent to opaque (see, e.g., [17]), but for the purpose considered here, which is thermal management, we aim to design a window that remains transparent to sunlight. We note that the considered radiative cooling windows (Figure 3(c) and (d)) are selective ones, which means that they emit or transmit radiation only within the ATW and reflect the rest of MIR light. Usually, selective radiative coolers are optimal for minimizing the equilibrium temperature, as they only exchange heat with the outer space without parasitic heat being emitted or absorbed in the process. On the other hand, horizontal broadband coolers (i.e., emitting in the whole MIR range) maximize cooling power due to the additional heat that lies at frequencies within the MIR but outside the ATW, and which is exchanged with the atmosphere [5]. However, in our scenario of windows in an urban environment, the limited view factor with the sky, along with the radiative exchanges with the opposite building and the ground that are generally hotter than the window itself, make broadband coolers a less attractive alternative (see Section III of Supplementary Material).

In Figure 3(e), we provide the calculated annual energy load due to heating (orange) and cooling (blue) for a building in Barcelona based on meteorological data from 2023. The considered building is equipped with one of the four aforementioned ideal windows or with conventional glass windows, for which the properties of SiO_2_ are considered using the refractive index from [31], [32]. The ideal properties for the four different type of windows pertain to unity absorptance, reflectance, and transmittance in the indicated spectral ranges. For example, in the case of a *solar heating* window, transmittance is unity across the visible and NIR ranges, and reflectance is unity across the MIR range. In the case of *emissive radiative cooling*, emissivity is considered to be unity within the ATW, and reflectance is unity for the rest of the IR range. The dimensions of the building are *L* = 20 m and *H* = 10 m, and the distance between buildings is set to *D* = 5 m (Figure 2). In order to perform a fair comparison between heating and cooling loads, we present the electrical cost for maintaining the room temperature constant, rather than the heat load *q*_load_ (Eq. (1)). We consider that heat is provided (or extracted) by a reversible heat pump with a seasonal coefficient of performance (SCOP) of 4 in heating mode and 5.6 in cooling mode, which corresponds to a A^+^ European energy class heat pump [33].

As is evident in Figure 3(e), the choice of window has a major impact on the heating and cooling loads. These loads can vary by a factor of 5–10 between the two most extreme cases. The effect is even greater in a building located in a rural location (i.e., when *D* → ∞), because the absence of nearby constructions (e.g., buildings) increases exposure to the sky, thus enhancing solar heating as well as radiative cooling.

As expected, the solar heating window has the smallest heating load, while the radiative cooling windows have the smallest cooling load. Interestingly, transmissive radiative cooling windows (Figure 3(d)) cool much better than their emissive counterparts (Figure 3(c)). This is because the latter introduces an additional thermal resistance between the room and the heat sink, which is the outer space at a temperature of 3 K. This thermal resistance does not occur in the case of transmissive coolers, as there is no element that absorbs or emits within the ATW in that case. The results of Figure 3(e) also demonstrate that optimizing a window for one season compromises its performance for others [15], as is expected. For example, although the solar heating window (Figure 3(a)) is the best solution for winter due to minimal heating demands, it is also the worst for summer, and vice-versa for the two cases of radiative cooling windows (Figure 3(c) and (d)). Eventually, the optimal static window for a building cannot be one that only operates well in one season, but rather one that can efficiently balance heating and cooling needs. In the absence of active tunability, the ideal window in terms of minimal overall electrical load is the one that completely reflects IR radiation (Figure 3(b)), as it significantly reduces the cooling need of buildings while containing the increase in heating needs. Using such windows instead of silica glass could reduce the overall electrical load of the building by more than 30 %. This demonstrates the importance of optimizing the radiative properties of windows for improved temperature regulation. We note that, in addition to the season it operates, the optimal choice for a static window also depends on the location considered and on the proximity of buildings in the surroundings (see Section II of Supplementary Material).

**Figure 4:**
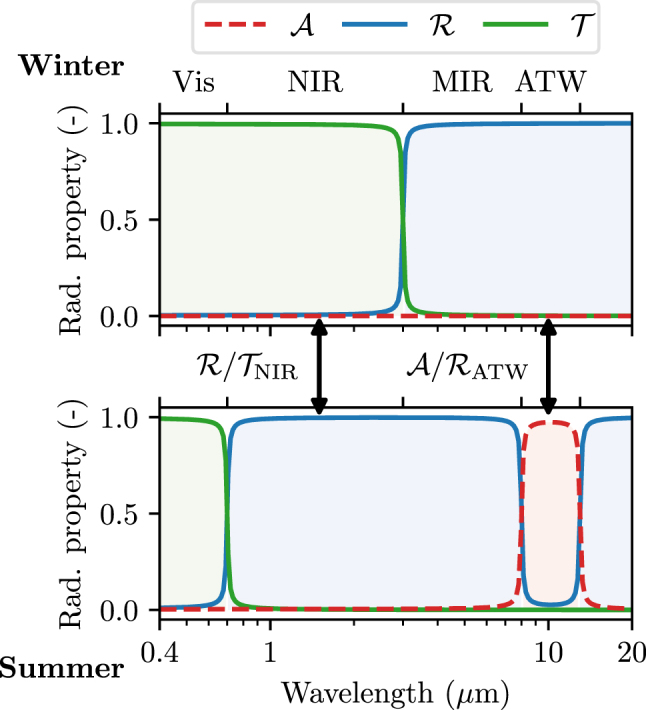
Winter and summer radiative properties of the aimed thermally smart window. Such a window should be able to switch between reflection and transmission in the NIR, and between reflection and emission in the ATW. It must also remain transparent in the visible and reflective in the rest of the MIR.

This section’s results suggest that the ideal thermally smart window to balance heating and cooling needs should switch between “solar heating” mode in winter and “transmissive radiative cooling” mode in summer. In practice, however, there is a scarcity of materials that could serve as a substrate for a transmissive radiative cooling window, which would remain transparent within the ATW while also being transparent at NIR and visible frequencies. Such a constraint would exclude SiO_2_ as a material platform, since SiO_2_ is opaque within the ATW. Incorporating other materials would significantly increase cost and restrict the results of this work to smaller-scale, niche applications. Due to this material constraint therefore, henceforth, we choose to design a thermally smart window that operates in an “emissive radiative cooling” mode in summer: although it is not optimal, it makes the use of a SiO_2_ substrate possible. Between summer and winter thereby, this realistically optimal window ought to exhibit tunable reflection (summer)/transmission (winter) in the NIR region and tunable reflection (winter)/emission (summer) in the ATW, as shown in Figure 4. Based on Figure 3(e), a building equipped with such windows with perfectly tunable properties will ideally use 6 GJ of energy for heating (as solar heating windows – see red box in Figure 3(e)), and 23 GJ of energy for cooling (as emissive radiative cooling windows – see blue box in Figure 3(e)), leading to an overall load of 29 GJ. This corresponds to a 50 % decrease in heating and cooling power with respect to the best static window, i.e., the one reflecting IR light, and to a 64 % decrease compared to conventional windows.

Finally, we note the small relative improvement of 25 % in cooling demand with an emissive radiative cooler (23 GJ, Figure 3(c)) as compared to an IR reflective window (31 GJ, Figure 3(b)). The cooling efficiency of the emissive radiative cooler is constrained by the lack of directionality in emission, leading to parasitic exchanges of heat between the building and its surroundings, including the opposite windows and the ground. Although solutions have been proposed in the literature to achieve directionality in vertical radiative cooling systems [14], they do not apply to windows due to the requirement of transparency and clarity at visible frequencies. Given the several constraints in the design of thermally smart windows, we will only consider isotropic emission in the following, keeping in mind the aforementioned limitation. Due to the limited improvement in cooling demand predicted with the emissive radiative cooler, one could argue that it is not worthwhile modulating emissivity in the ATW, and that one should rather focus on NIR modulation. While this is theoretically reasonable, we show below that the range of realistically available tunable materials makes the efforts for emissivity modulation within the ATW worthwhile. In fact, ATW emissivity modulation can be greater than NIR reflectivity modulation, making it reasonable to combine the two solutions in a single smart window.

## 3 Conceptual windows

In this section, we aim to determine photonic structures that can approach the aforementioned optimal radiative properties. First, we study independently the two spectral ranges where active modulation is required for thermally smart windows, namely the NIR and ATW. In Section 3.3, we combine the two solutions. As shown, the mechanisms identified and discussed henceforth pertain to phase-changes, electrostatic gating, and liquid crystals, due to their increasing applicability and large-scale potentials. We note that, although other mechanisms can serve as means to actively modulate the photonic properties of windows, such as mechanical and wetting that can lead to sizable modulation [34], we exclude them from the current study to restrict ourselves to systems with no moving parts. Unless otherwise stated, the radiative properties of structures are computed using the free software *Reticolo* [35], which performs rigorous coupled wave analysis.

### 3.1 ATW modulation

**Figure 5:**
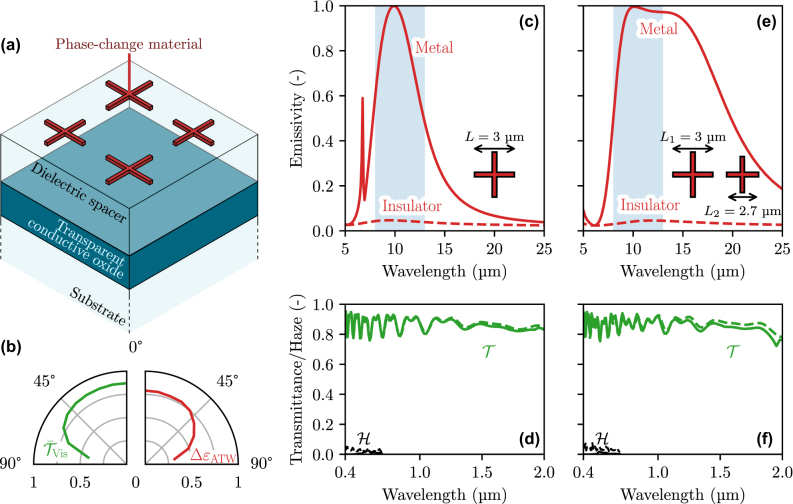
Performance of a thermally smart structure modulating ATW/MIR radiation, composed of PCM resonators placed in an emitter/spacer/reflector configuration to enhance emission. (a) Schematic of the structure. (b) Directional mean visible transmittance and ATW emissivity modulation of the structure, between its two states. (c) MIR emissivity and (d) visible and NIR transmittance of the selective emitter at normal incidence. (e) MIR emissivity and (f) visible and NIR transmittance of the broadband emitter at normal incidence.

In the past few years, there has been increasing interest in developing radiative coolers that remain transparent at visible wavelengths, with static [15], [36] as well as tunable MIR properties using VO_2_ [26], [27], [28], [37]. Recent architectures combine a MIR emitter with a dielectric spacer and an IR back reflector, in a manner similar to a Salisbury screen [38], to enhance emissivity within the ATW through constructive interferences. However, these solutions lack selectivity, thereby emitting within the whole MIR range, which becomes detrimental during hot periods, as explained above. They also present limited transparency at visible frequencies, thereby reducing their relevance for windows applications. Here, to achieve spectral selectivity within the ATW, we introduce cross-shaped resonators as substitutes to previous emitters (thin film [26] or square-shaped resonators [37]), while also considering the basic principle of a generalized Salisbury screen as represented in Figure 5(a) with the emitter-spacer-reflector geometry. The cross resonators can be made of any phase-change material (PCM) that can undergo a reversible metal-insulator transition. Luckily, recent results have demonstrated sufficient and robust MIR modulation with some phase-change materials, for example VO_2_ [37], [39], [40], In_3_SbTe_2_ (IST) [41], [42], or GeSbTe (GST) for instance [43]. While VO_2_ is the most common choice due to its low critical temperature, other materials such as IST or GST, which are nonvolatile and could thus allow for on-demand switch, are also appropriate. In their metallic phase, the cross resonators can act as selective emitters [44] due to localized surface plasmon excitations (see Section IV of Supplementary Material), the resonant wavelength varying linearly with the length of the cross’ arms [45]. In addition, the shape of a cross inherently reduces the surface coverage compared to square resonators, leading to higher transparency at visible wavelengths. The spacer should be made of a material that is transparent from the visible to the MIR, while the back-side reflector of the generalized Salisbury screen is composed of a transparent conductive oxide (TCO, e.g., ITO or AZO).

For simplicity, we consider the substrate to be made of the same material as the spacer and, thus, to be transparent across the visible, NIR, and MIR spectral ranges. In reality, the spacer layer could absorb in the MIR (for instance if made of SiO_2_); this would have no impact in the device’s performance, as the TCO is opaque for such wavelengths. We suppose that the dielectric spacer and the substrate have a refractive index of 1.5 over the whole wavelength range, which is close to the value of the refractive index of several dielectrics, e.g., BaF_2_ or CaF_2_, ZnSe, or PMMA. In theory, the PCM should absorb radiation in the visible (and potentially in the NIR) due to interband transitions. However, with a surface coverage close to 3 % in the scenario analyzed, we consider that the interband contribution (and the related visible and NIR absorption) can be neglected. To keep the results general, instead of considering the properties of specific materials, we introduce a simple Drude oscillator model to describe the metallic phase of the PCM as well as the dielectric response of the TCO:

(2) $$\varepsilon_{\text{Drude}}=\varepsilon_{\infty }-\frac{\omega_{\text{p}}^{2}}{\omega \left(\omega +i\gamma \right)},$$

where *ɛ*_∞_ represents the high-frequency dielectric constant, *ω*_p_ is the plasma frequency, and *γ* is the damping coefficient. For both the TCO and the PCM, *ɛ*_∞_ is set to 4, close to that of materials such as indium tin oxide (ITO) [46], VO_2_ [47], and IST [41]. *γ* is equal to 0.1 eV for the PCM, and to 10 meV for the TCO. The low-*γ* rate of the TCO is essential for achieving highly efficient windows (see Section V of Supplementary Material where the effect of *γ* is investigated). *ω*_p_ is set to 5 eV for the PCM (close to that of IST [41] for the example) and to 1 eV for the TCO, respectively. We take the PCM refractive index to be constant and equal to 3 in the insulating phase. In addition, the PCM is assumed to be nonvolatile.

The calculations are carried out considering that incident radiation comes from the outside (i.e., from the side of the PCM resonators in Figure 5(a)). We perform an optimization of the device’s geometric parameters using the MATLAB function *patternsearch*, by minimizing the figure of merit (FOM) defined as:

(3a) $$FOM=\frac{1}{8\sqrt{3}}\underset{m}{\sum }\underset{\lambda_{r}}{\sum }\|X_{m,\lambda_{r}}-X_{m,\lambda_{r}}^{\text{goal}}{\|}_{2},$$

(3b) X=(A,R,T).

In these expressions, A , R and T are the absorptance, reflectance, and transmittance of the window. “*m*” and “*λ*_*r*_” represent, respectively, the two seasonal modes of the window and the four different wavelengths ranges of interest (visible, NIR, ATW, and rest of the IR). The goal for each mode and each spectral range is set accordingly to Figure 4, except in the NIR where the window should always be transparent to make the device integration with NIR-modulating structures straightforward, as we show in the following sections. The factor $1/8\sqrt{3}$ normalizes the FOM to a value between 0 and 1, with 0 being “exactly the goal” and 1 “exactly the opposite of the goal.” Finally, the value of each radiative property *χ* in a wavelength range is defined as:

(4) $$\chi_{\lambda_{\text{r}}}=\frac{\underset{\lambda_{\text{r}}}{\int }\chi \left(\lambda \right)P\left(\lambda ,T\right)d\lambda }{\underset{\lambda_{\text{r}}}{\int }P\left(\lambda ,T\right)d\lambda },$$

where P(λ,T) is the Planck distribution, the temperature *T* being that of the Sun (5780 K) for visible and NIR radiation, and that of the ambient temperature (300 K) for MIR radiation. The following results are obtained considering unpolarized incident light.

First, we consider that all cross-shaped resonators are similar. Our optimization leads to a structure with a 1 µm TCO layer, a 1.4 µm dielectric spacer, and 75 nm thick resonators with 3 µm arm length and 100 nm arm width. The distance between two resonators is equal to 1.8 µm. The resulting radiative properties are shown in Figure 5(c) and (d). The structure displays remarkably strong emissivity modulation in the MIR, with high selectivity: the emissivity varies from 4 % to 84 % in the ATW (Δ*ɛ*_ATW_ = 79 %) but remains low in the rest of the MIR (3 % and 26 % in the insulating and metallic phase, respectively). The structure is also highly transparent to solar radiation, with a mean transmittance in the visible and NIR ranges of 85 % in both phases.

The structure can also be adapted to achieve MIR emission for a broader MIR range by varying the characteristic length of the resonator, each individual resonance contributing to a broader emission [48]. This is required to achieve high cooling rate in horizontal radiative cooling for instance [5]. The optimization algorithm then yields crosses of two different sizes of 3 and 2.7 µm long arms, respectively, while their width and their separation distance are reduced to 50 nm. The spacer in this case is 1.8 µm thick. As expected, this strongly improves the emissivity modulation in the whole MIR (see Figure 5(e)), which switches between 3 % in one phase and 60 % in the other (Δ*ɛ*_MIR_ = 56 %) while achieving 86 % transparency to solar light (see Figure 5(f)). In comparison, previous experimental studies achieved visible transmittance up to 60 % and MIR modulation between 30 and 40 % using VO_2_ [26], [27], [37], while theoretical analyses obtained transparency at visible wavelengths of the order of 70 % and ATW emissivity modulation up to 84 %, but without appreciable selectivity [28], [49]. Thus, the structures considered here that are well-within nanofabrication capabilities (see, for example [42], for IST resonators) achieve excellent emissivity modulation over a broad angular range for either selective or broadband emission, as shown in Figure 5(b).

### 3.2 NIR modulation

As was shown in Figures 3 and 4, to achieve optimal active temperature regulation, a window is required to switch between a transmissive and reflective phase at NIR frequencies. Previous works have considered thermochromic materials such as VO_2_ [23], [26], [50], or electrochromic materials (e.g., ITO, Al-doped ZnO, or WO_3_) in the form of thin films [51] or plasmonic nanocrystals [22], [24], [52], [53], [54]. All these solutions, however, share the disadvantage of being *absorptive* in at least one phase at NIR frequencies. This hinders their applicability for thermal management. In addition, the majority of previously considered solutions also absorb at visible frequencies. An alternative approach is the use of ITO and other TCOs, since these can be modulated through electrostatic gating [55], [56]. While such materials suffice for NIR modulation in imaging, telecommunications, and other applications, the degree of modulation typically achieved is insufficient for thermal management. This originates from the very small Debye length of TCOs, within which modulation of carrier density occurs, especially when considering the very high carrier concentration needed to make them reflective close to visible frequencies. For this reason, and despite the wide-spread use of electrostatic gating as a mechanism for tuning photonic properties, we exclude TCOs from the options for NIR modulation. Some other works have studied structures that are simultaneously tunable at visible and NIR frequencies [57], [58], therefore, lacking transparency to visible light in one of the two states. In the following, we consider two potential pathways of tuning a window between transmissive and a reflective state at NIR frequencies: using insulator-to-insulator PCMs, and cholesteric liquid crystals.

Let us first consider insulator-to-insulator PCMs. Such materials are semiconductors. To ensure transparency at visible frequencies, ideally, their bandgap should lie in the UV range (or at least close to it). Some notable examples of insulator-to-insulator PCMs include GeSbSeTe (GSST) alloys or Sb_2_S_3_ and Sb_2_Se_3_ alloys [59]. The most transparent material out of these is Sb_2_S_3_, with a bandgap energy of 1.6 eV and 2.2 eV in its two phases, corresponding to wavelengths of 0.8 and 0.55 µm, that, unfortunately, both lie in the visible range [60]. Due to this non-negligible absorption within the visible spectral range, we consider two solutions: to either keep the PCM film optically thin or to pattern it to limit the surface coverage.

**Figure 6:**
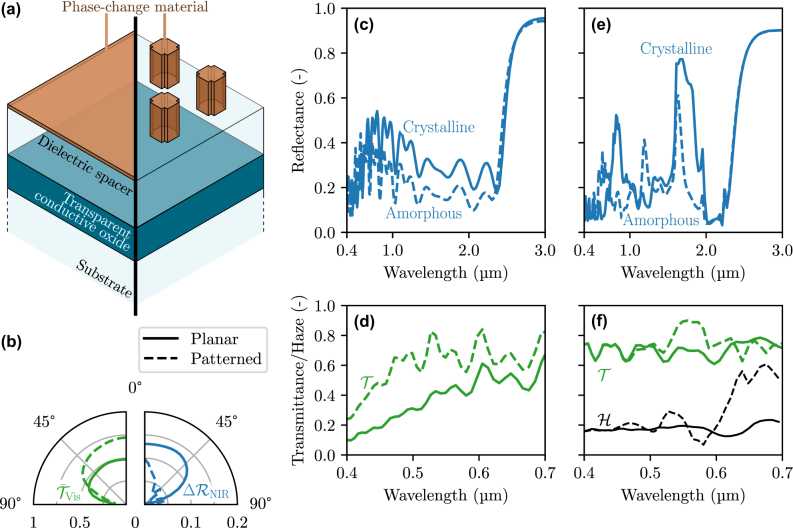
Performance of a thermally smart structure modulating NIR radiation based on IR-transparent PCMs, either in the form of thin films or resonators. (a) Schematic of the structure. (b) Directional mean visible transmittance and NIR reflectance modulation of the structure, between its two states. (c) NIR reflectance and (d) transmittance at visible frequencies of the smart structure made of a thin-film PCM at normal incidence. (e) NIR reflectance and (f) visible transmittance of the smart structure made of PCM resonators at normal incidence.

We have investigated the potential of these two solutions based on Sb_2_S_3_, using the same spacer/TCO/substrate structure as for MIR modulation in the previous section (see Figure 5(a)) for better interoperability. The complete structure is shown in Figure 6(a), with their directional properties in panel (b) and their spectral properties in panels (c–f). In this case, the FOM described in Eq. (3) is restricted to the visible and NIR ranges, with the objective properties being set according to Figure 4. The optimal film is 80 nm thick, while the optimal resonator is 1 µm thick with arms that are 0.5 µm long and 0.3 µm wide. The distance between resonators is equal to 1.1 µm. Both solutions have their advantages and drawbacks. While the thin-film solution leads to significant reflectance modulation for angles of incidence up to 60° as shown in Figure 6(b) and (c), and being simple to fabricate with thin-film deposition, it shows only limited transparency at visible frequencies (49 % at normal incidence – Figure 6(b) and (d)). Nanostructuring Sb_2_S_3_ significantly improves transmittance at visible frequencies (72 % – Figure 6(b) and (f)) as expected but makes the structure hazy while the NIR modulation is also compromised. As shown in Figure 6(f), roughly 20 % of the incoming light is scattered during transmission, and as shown in Figure 6(b) and (e), the resonators significantly reduce the capability to modulate NIR radiation for oblique incidence. This is especially important as sunlight enters the window obliquely. In both the case of a thin film and nanoresonators, the reflectance modulation remains rather modest, reaching respectively 13 % and 9 % at normal incidence for the thin-film and nanostructured solutions. This arises from the limited refractive index contrast of Sb_2_S_3_ that does not exceed 0.5 at most, as well as the non-negligible absorption of this material at visible frequencies. It becomes evident that additional research and development of more appropriate transparent insulator-to-insulator PCMs could significantly improve these results.

**Figure 7:**
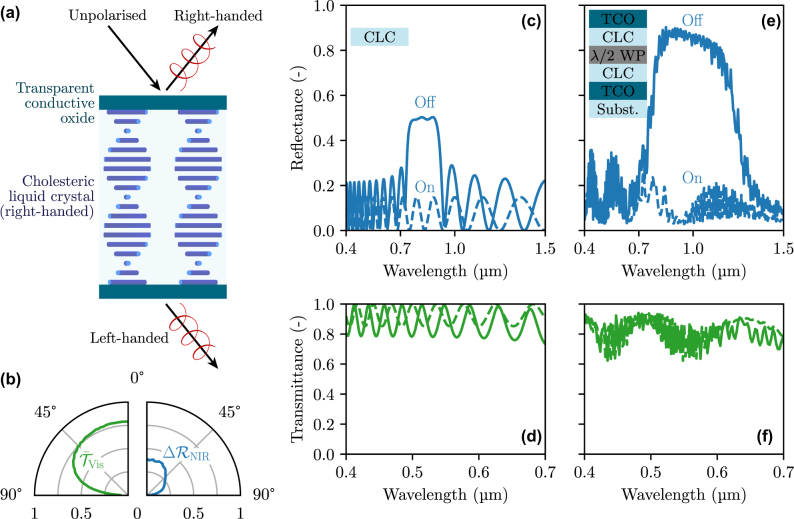
Performance of a thermally smart structure modulating NIR radiation based on cholesteric liquid crystals (CLCs), which act as circular-polarization-selective photonic crystals. (a) Schematic of the structure. (b) Directional mean visible transmittance and NIR reflectance modulation of the double CLC structure shown in panel (e), between its two states. (c) NIR reflectance and (d) visible transmittance of a single CLC. (e) NIR reflectance and (f) visible transmittance of the smart structure made of two identical CLCs separated by a half-waveplate for polarization-independent response, and placed between two TCO electrodes to enable the switching.

A second solution for modulating NIR reflectance is with cholesteric liquid crystals (CLCs), also termed chiral nematic liquid crystals. In CLCs, polymers are organized in helical structures along a principal axis, making the structure anisotropic and causing its in-plane refractive indices to vary periodically with depth. This makes them act as a photonic crystal, for light with polarization handedness similar to that of the crystal, while they remain transparent for the other circular polarization, as represented in Figure 7(a). The resonant wavelength of the structure can be tuned by controlling the concentration in chiral dopant added during fabrication [21]. In addition, the selective photonic crystal behavior can be switched off by applying a bias through the CLC, which will reorient the polymers in the direction of the field. This active tunability makes CLCs a promising candidate for NIR modulation [61].

We simulate the properties of CLCs using an in-house open-source solver based on the transfer matrix method for anisotropic structures [62]. As an example, we show the reflectance and transmittance of a single CLC in Figure 7(c) and (d), the CLC being 2.5 µm thick and the helical structure pitch being set to 0.5 µm. Its ordinary and extraordinary refractive indices are set to 1.5 and 1.8, respectively, in line with the properties of real liquid crystals [63]. The photonic bandgap clearly appears in the “Off” state (i.e., unbiased) between 0.7 and 0.9 µm, the reflectance only reaching 0.5 due to the polarization selectivity of the structure. When a voltage is applied (“On state”), the reflectance reduces to roughly 10 %. We also consider a more complete structure with transparent conductive materials at the boundaries of the CLC to make it possible to apply a voltage, as shown in Figure 7(a). We also consider a pitch gradient, which can be induced during polymerization to make the photonic bandgap wider [21], and we combine two identical CLCs with a half-waveplate in between them, to generalize the device’s operation to both circular polarizations. The properties of such a structure, considering 10 µm CLCs with 1 % pitch gradient, are shown in Figure 7(e) and (f). The TCO layers are 0.5 µm thick, and their refractive index is the same as the one presented in Section 3.1. The ordinary and extraordinary refractive indices of the waveplate are 1.54 and 1.55, respectively, close to those of quartz. Although the TCO layers induce some absorption and reflection in the NIR, the structure achieves 33 % NIR reflectance modulation at normal incidence, while ensuring high transparency in the visible range (75 %). These properties are well-maintained up to 45° incidence angles, as shown in Figure 7(b).

To increase NIR modulation, one ought to extend the spectral range of the photonic bandgap of the CLCs. This is possible by increasing the pitch gradient or the CLC thickness, as long as the operation of the half-waveplate is unperturbed – one can utilize two CLCs with opposite chirality to eliminate the need for a half-waveplate. Another solution to increase the spectral range of the photonic bandgap would be to combine two CLC/waveplate/CLC structures with different pitch on top of each other. However, all the solutions involving an increase in thickness will also cause a rise in the electrical switching cost. If this energy cost remains mostly negligible for simple structures [64], it could become significant for more complex structures, and the trade-off between the structure modulating properties and its energy and economic viability should be considered.

### 3.3 Complete thermally smart window

In Section 3.1, we presented a solution for modulation of thermal emissivity within the ATW, essential for efficient cooling in summer, using cross-shaped resonators made of a metal-to-insulator PCM. In Section 3.2, were presented approaches for modulation of NIR reflectance, essential for efficient rejection of solar heat in summer and solar heating in winter. Here, we demonstrate that integrating these solutions in a single device, to achieve simultaneous dynamic control over the MIR and ATW spectral ranges, can be straightforwardly implemented.

**Figure 8:**
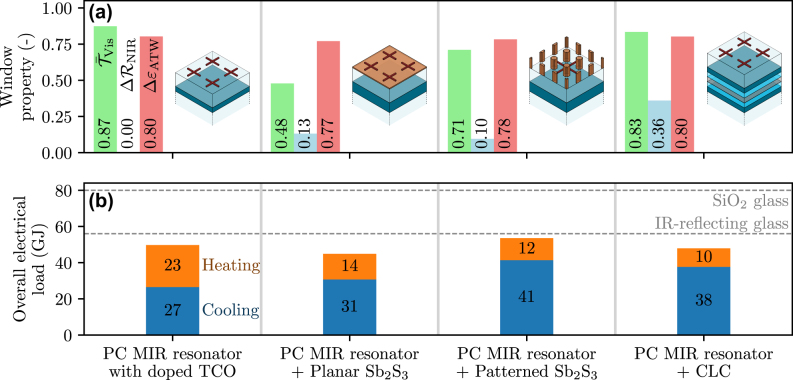
Performance of the different solutions investigated as thermally smart windows. For each solution, we represent in (a) their radiative properties, and in (b) the subsequent energy demand of a building when equipped with such windows. The values obtained for silica glass and for reflecting glass, which were reported in Figure 3, are shown in dashed line. These results are obtained considering that the radiative properties are independent of the direction.

Considering the planar Sb_2_S_3_ layer presented in Section 3.2 for NIR modulation, this layer’s thickness is negligible compared to that of the spacer (Figures 5(a) and 6(a)), hence, can be placed directly below the metal-to-insulator PCM crosses. Considering, on the other hand, the nanostructured Sb_2_S_3_ solution of Figure 6(a), the Sb_2_S_3_ resonators have a periodicity that is one third of that of the metal-to-insulator PCM crosses; hence, they can be placed in between each other. As for the CLC solution (Figure 7(a)), the CLCs may be positioned between the TCO and the substrate, the TCO acting as one of the CLC’s electrodes. In all aforementioned solutions integrating NIR and ATW modulation, the spectral response of the complete device does not change significantly compared to the individual devices discussed in Figures 5–7; hence, we do not show them for the sake of brevity. Rather, we compare in Figure 8 the global radiative properties of these different solutions, along with their impact on the energy consumption of a building when used as thermally smart windows, using the heat balance model detailed in Section 2. For simplicity, the radiative properties are considered to be independent of the angle of incidence or emission, and equal to the properties calculated at normal incidence, thus slightly overestimating the modulation capability of the structures.

As a reference, we compare these solutions with a structure constrained to MIR modulation (Figure 5), which is shown in the left of Figure 8 and labeled as “PC MIR resonator with doped TCO” (“PC” standing for phase-change). Since we have already demonstrated in Section 2 that reflecting NIR radiation is actually more impactful (in summer) for thermal management that allowing NIR radiation to be transmitted (in winter) – for the latitude of Barcelona due to the high cooling needs – we consider the TCO layer of this particular structure (see Figure 5(a)) as highly doped (*ω*_*p*_ = 10 eV) and thinner (250 nm) to make it reflective in the NIR, thus ensuring that this solution is as efficient as possible.

Several conclusions can be drawn from the results in Figure 8. First, one notices that the ability to modulate the radiative properties within the NIR and ATW ranges is almost unaffected by their integration in a single device, varying by less than 4 % across all devices. By contrast, one can notice that the addition of NIR modulation only brings limited improvement in terms of energy needs. This is due both to the value of Δ*R* itself and to the low NIR reflectance achieved by the architecture in its two states (see Figures 6 and 7). Out of the three smart windows, the one involving planar Sb_2_S_3_ leads to the lowest electrical load with a reduction of 10 % compared to the MIR-tunable smart window, although this is partly due to its limited transparency at visible frequencies during the hot months. This solution is also the simplest to fabricate and the thinnest of all three. In comparison, the increased transparency and decreased NIR modulation of the solution based on structured Sb_2_S_3_ lead to increased electrical load that occurs in summer due to less efficient cooling. As for the CLC-based smart window, it achieves the best transparency at visible frequencies as well as maximal tunability.

All the solutions investigated surpass the best static window (the IR-reflecting window, shown in dashed line in Figure 8(b)) in terms of thermal management, since they all present significantly smaller overall electrical load. In fact, the building’s electrical load with all solutions proposed is tens of gigajoules lower as compared to conventional glass, corresponding to a decrease in the energy demand for heating and cooling between 34 % (for the solution with patterned Sb_2_S_3_) to 44 % (for the one involving planar Sb_2_S_3_).

## 4 Outlook

This work underlined the potential of thermally smart windows that can modulate their radiative properties across the whole infrared range for thermal management considerations. As we demonstrated, such windows can, ideally, reduce the energy demand associated to heating and cooling of a building in Barcelona by almost two thirds when compared to silica glass. If the results in Figure 3 highlighted the *theoretical* capabilities of thermally smart windows considering ideal materials, those in Figure 8 underscored the practical feasibility of these solutions, yielding up to 44 % of estimated energy savings using PCMs.

To maximize temperature regulation, a thermally smart window should be able to tune its optical response simultaneously in the near-IR (between transmission and reflection) and in the mid-IR (between reflection and emission). This motivated the design of different structures for NIR and MIR modulation, based on phase-change materials and cholesteric liquid crystals. We proposed structures that exceed 80 % emissivity modulation, while ensuring high transparency at visible frequencies. We showed that modulation of NIR reflectance has the greater potential for thermal management, by controlling the influx of solar radiation that carries a significant amount of heat (see Figure 1). Nonetheless, we discussed that, in practice, the currently available solutions in terms of materials properties yield limited reflectance tunability, which results in a significantly smaller performance improvement as compared to the realistically possible options for modulating the MIR properties of windows (Figure 5).

Thereby, an important conclusion of this work is that material development that warrants tunable and broadband radiative properties within the NIR is key for the development of optimally efficient thermally smart windows and can lead to highly disruptive progress in the field of thermal management. We note that encouraging results are already being reported, for example, with research on emerging PCMs that is rapidly growing. As an example, the interest in Sb_2_S_3_ has only risen in the past few years, with promising and relevant results reporting active tunability [60], [65], [66]. An important objective in the research of PCMs should be to identify NIR-transparent PCMs with larger refractive index contrast, or PCMs that are transparent (nonabsorbing) at visible frequencies. Regarding CLCs, since their ability to modulate their NIR properties is mostly limited by the width of their photonic bandgap, several solutions are becoming available, as mentioned in Section 3.2. However, this comes at the cost of additional device complexity; hence, energy expenses for electrically switching the CLCs should be considered carefully. Thereby, we believe that further investigation is required to clarify the full potential and feasibility of CLCs as a solution for thermally smart windows.

## Supplementary Material

Supplementary Material Details
