# Supplementary material for: Energy-efficient thermally smart windows with tunable properties across the near- and mid-infrared ranges
Source: Nanophotonics. 2025 Jun 17;14(23):4259–72. doi: 10.1515/nanoph-2025-0219 (PMC12617719; doi:10.1515/nanoph-2025-0219)
Supplement: Supplementary file 1 — Supplementary Material Details [file j_nanoph-2025-0219_suppl_001.pdf]

Supplementary Material

Julien Legendre and Georgia Papadakis*

Energy-efficient thermally smart windows with tunable properties across the near- and mid-infrared ranges

Contents

S1 Physical model for energy demand estimation 1

S2 Influence of location on the energy demand of buildings 4

S3 Broadband vs. selective radiative cooling 6

S4 Details on the resonant behavior in the atmospheric window 7

S5 Impact of metallic losses on tunable window performance 8

S1 Physical model for energy demand estimation

As discussed in the main article, the energy demand of a building for heating and cooling purposes can be expressed as the sum of five distinct contributions:

$$q_{\text{load}} = q_{\text{sun}} + q_{\text{sky}} + q_{\text{ground}} + q_{\text{building}} + q_{\text{nonrad}}. \tag{1}$$

The time-varying solar illumination on the different façades is obtained from the Python package *PVLIB* [5], and includes the multiple reflections between the two buildings (supposed to be perfectly diffuse), along with the shadow cast by one building on the other. This leads to

$$q_{\lambda,\text{sun}} = \frac{f\mathcal{T}_{\text{window}}}{1-(\text{VF}_{\text{b}}\mathcal{R}_{\text{b}})^2}\left(f_I q_{\lambda,\text{dir}} + \text{VF}_{\text{b}}\mathcal{R}_{\text{b}} f_I^{\text{opp}} q_{\lambda,\text{dir}}^{\text{opp}}\right) + \text{VF}_{\text{sky}}\frac{f\mathcal{T}_{\text{window}}}{1-\text{VF}_{\text{b}}\mathcal{R}_{\text{b}}} q_{\lambda,\text{dif}}, \tag{2}$$

where $\mathcal{T}_{\text{window}}$ is the window transmittance, and f represents the fraction of the area that is covered by the windows, set to 0.5 in the article and in the following. f_I is the fraction of the area being illuminated, which depends on the position of the Sun and of the shading caused by the opposite building. It is obtained by geometrical means. $q_{\lambda,\text{dir}}$ and $q_{\lambda,\text{dif}}$ are the direct and diffuse contributions obtained from *PVLIB* using the atmospheric properties summarized in Table S1. Superscript *opp* indicates that the Sun radiates towards the opposite surface, the radiation being eventually transmitted to the room after reflection. $\mathcal{R}_{\text{b}}$ is the effective building reflectance:

$$\mathcal{R}_{\text{b}} = f\mathcal{R}_{\text{window}} + (1-f)\mathcal{R}_{\text{wall}}. \tag{3}$$

Tab. S1: Atmospheric properties considered for computing the solar irradiance with package *PVLIB*.

Physical quantity	Precision	Value	Unit	Source
Pressure	Direct use of time-dependent experimental data	-	Pa	[1]
Water vapor content	Assumed to vary sinusoidally over the year (min. on January 1st)	0.5 - 3	cm	[2]
Ozone content	Assumed to be constant, estimate of mean value in nearby stations	0.35	atm.cm	[3]
Aerosol turbidity at 500 nm	Assumed to be constant, use of the mean value	0.14	-	[4]

Julien Legendre, ICFO-Institut de Ciencies Fotoniques, The Barcelona Institute of Science and Technology, Castelldefels, Barcelona 08860, Spain; ORCID nº 0000-0001-7316-1954.
***Corresponding author: Georgia Papadakis,** ICFO-Institut de Ciencies Fotoniques, The Barcelona Institute of Science and Technology, Castelldefels, Barcelona 08860, Spain; ORCID nº 0000-0001-8107-9221; georgia.papadakis@icfo.eu

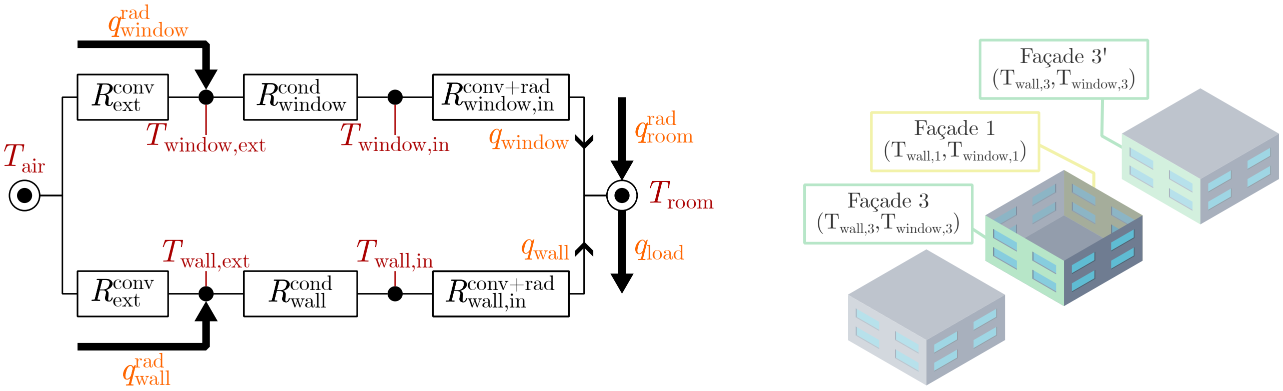

(a) **(b)**

Fig. S1: (a) Equivalent electrical circuit considered for the calculation of the heat exchanged between the windows, the walls and the room. (b) Illustration of the periodicity of the urban environment. To compute the non-radiative contribution at façade 1 (yellow), one need the temperatures of façade 1 as well as those of the facing façade 3' (green). Because of the periodicity of the urban environment, façades 3 and 3' have exactly the same temperatures, and the wall and window temperatures at façade 3' can be used to compute the non-radiative contribution at façade 3.

VF stands for view factor. Because two opposite façades are identical and parallel, the view factor between two buildings $\mathrm{VF_b}$ can be computed analytically. Note that other buildings (placed in diagonal, for instance) could also exchange heat with the one we study or cast a shadow onto it, but are neglected for simplicity. The sky and the ground then share the same view factor, leading to $\mathrm{VF_{sky}} = \mathrm{VF_{ground}} = (1 - \mathrm{VF_b})/2$.

We suppose that the ground is a black body, leading to

$$q_{\lambda,\mathrm{ground}} = \mathrm{VF_{ground}} \frac{f\mathcal{T}_{\mathrm{window}}}{1 - \mathrm{VF_b}\mathcal{R}_\mathrm{b}} (\mathcal{P}(\lambda, T_{\mathrm{ground}}) - \mathcal{P}(\lambda, T_{\mathrm{room}})), \tag{4}$$

$\mathcal{P}_\lambda(T_{\mathrm{room}})$ being Planck's distribution. The ground temperature T_{ground} is supposed to be equal to air temperature, whose hourly variation is provided by the *Meteostat* package [1].

The expression is mostly similar for the atmospheric contribution. The atmosphere is supposed to have a unitary emissivity for all wavelengths except between 8 and 13 μm, where its emissivity is supposed to be constant and equal to 0.2 to account for its transparency (estimated from [6] using the methodology presented in [7]). This transparency decreases the emission of the atmosphere, but does not impact the energy lost by the building through emission, as radiation that is not absorbed by the sky simply escapes towards space instead of being absorbed. Consequently, the atmospheric contribution is

$$q_{\lambda,\mathrm{sky}} = \mathrm{VF_{sky}} \frac{f\mathcal{T}_{\mathrm{window}}}{1 - \mathrm{VF_b}\mathcal{R}_\mathrm{b}} (\varepsilon_{\mathrm{sky}}\mathcal{P}(\lambda, T_{\mathrm{air}}) - \mathcal{P}(\lambda, T_{\mathrm{room}})). \tag{5}$$

To be completely rigorous, the emission coming from outer space should also be included, but it can be neglected because of the low temperature of space (3 K).

The heat transfer between the buildings and the room q_{building} is in fact a sum of four different terms, as radiation can be exchanged with the wall and the windows of both facing façades. For the sake of brevity, the complete expressions will not be provided here, being mostly similar to the previous ones. Finally, the non-radiative contribution is expressed as

$$q_{\mathrm{nonrad}} = f h_{\mathrm{window}}(T_{\mathrm{window,ext}} - T_{\mathrm{room}}) + (1 - f)h_{\mathrm{wall}}(T_{\mathrm{wall,ext}} - T_{\mathrm{room}}), \tag{6}$$

with h representing the heat transfer coefficient. To compute this contribution, the window temperature $T_{\mathrm{window,ext}}$ and the wall temperatures $T_{\mathrm{wall,ext}}$ on the outer façade must be calculated. This is done using heat balance between convection with the outside air, conduction through the façade and exchange of radiation with the sky, the ground and the opposite building, as schematized in Fig. S1a. The thermal resistances are obtained considering:

– walls made of 20 cm of concrete (with thermal conductivity $\kappa = 1.5$ W.m^{-1}.K^{-1}) and 5 cm of isolating material ($\kappa = 0.05$ W.m^{-1}.K^{-1});
– windows made of 1 cm of glass ($\kappa = 0.8$ W.m^{-1}.K^{-1}) with a 1 mm layer of air ($\kappa = 0.026$ W.m^{-1}.K^{-1}) inside;
– a convective heat transfer coefficient of $h = 10$ W.m^{-2}.K^{-1} outside;

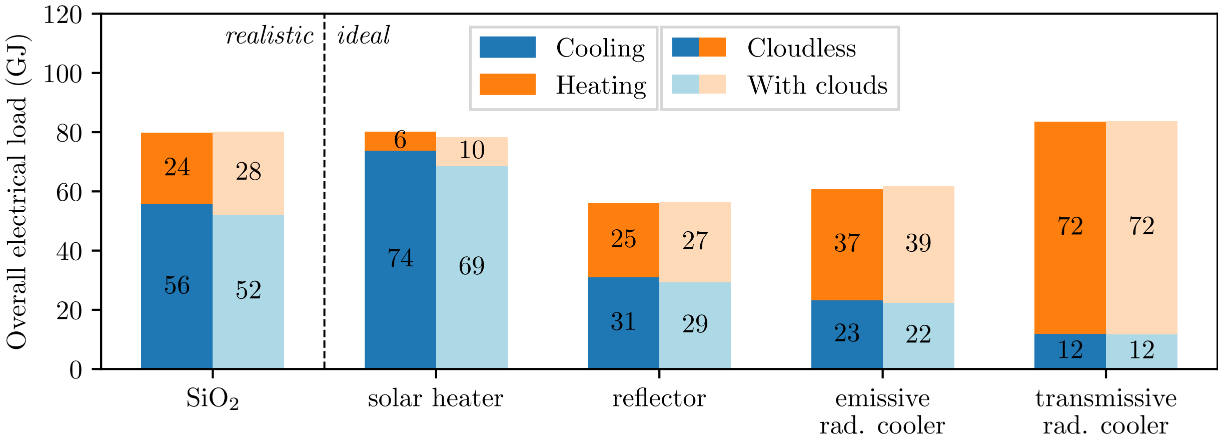

Fig. S2: Impact of the consideration of cloud coverage on the yearly energy demand of buildings in Barcelona.

– a convective heat transfer coefficient of $h = 5$ W.m^{-2}.K^{-1} inside;
– an emissivity for the wall equal to 0.7 in the visible and the NIR, and to 0.95 in the MIR [8].

For simplicity, because the difference of temperature between the room and the inner surface of the façade is small, we consider heat transfer by radiation to be linear with the temperature difference. We add to the convective heat transfer coefficient h a radiative contribution $h_{\rm rad}$, which is around 5 W.m^{-2}.K^{-1} for black bodies around 300 K. Since the façade is not a black body, $h_{\rm rad}$ must be corrected. Using that one third of the emission of a black body at 300 K is located in the ATW, we get the approximate expression $h_{\rm rad} = 5(1/3 \cdot \varepsilon_{\rm ATW} + 2/3 \cdot \varepsilon_{\rm MIR-rest})$ W.m^{-2}.K^{-1}. In addition, we suppose that no heat is transferred through conduction between the window and the wall, and that all the radiation coming from outside and absorbed in the façade is absorbed at the external surface (so that it directly impacts the temperature at the surface, as shown in Fig. S1a). Because radiation is exchanged between windows and walls, not only from opposite buildings but also from the same building (after reflection on the opposite building), the four temperatures of the windows and walls of both façades are coupled, and are all different due to the asymmetric solar irradiation on each façade. The four-equation system is solved using the *root* function of the *Scipy* Python package. Once they are known, the non-radiative contribution $q_{\rm nonrad}$ can be computed from Eq. (6). Due to the periodicity of the urban environment, the temperatures of the facing façade (façade 3' in Fig. S1b) are exactly those of the opposite façade of the building of interest (façade 3 in Fig. S1b). They can thus be used to compute the non-radiative contribution at this other façade simultaneously, reducing the computational time.

Last, note that Eqs. (2) and (5) hold only for clear skies, the presence of clouds reducing both solar heating and radiative cooling. Still, for a building in Barcelona, the energy demand is almost not impacted by cloud coverage, as seen in Fig. S2: this is why the sky is supposed to be cloudless in the main article. To estimate the impact of cloud coverage in Fig. S2, we used the weather condition code ζ from *Meteostat* data [1], which we translated into two parameters:
– the cloud coverage itself, which corresponds to the fraction of radiation that is attenuated by clouds. We set it to 0 when $\zeta = 1$ or 2 (clear sky), to 0.5 when $\zeta = 3$ (cloudy) and to 1 when $\zeta \geq 4$ (overcast, rain, storm, etc.).
– the transmission coefficient of clouds, which we set to 0.3 when $\zeta = 3$ (cloudy) or 4 (overcast), and to 0.15 for larger ζ (rain, storm, etc.). These have been estimated from the cloud optical depth presented in [9] and from the relation between optical depth and transmission coefficient reported in [10]. In addition, all the radiation that is attenuated by clouds is supposed to be diffuse after transmission.

Because cloud attenuation is a secondary phenomenon in the system we study, we preferred to keep its modelling simple. However, note that the model above is approximate, and that refining it would require a more detailed analysis of the influence of clouds.

S2 Influence of location on the energy demand of buildings

In the main document, an urban environment was considered with a distance between two buildings equal to 5 m. It is key to have in mind that the presence of buildings in the surroundings of the building of interest impacts significantly its energy needs. To illustrate this, we show in Fig. S3 the results obtained for a building at the latitude and with the climate of Barcelona in a rural environment, i.e. with no buildings around. Such a configuration makes the view factor between the façade and the sky larger, especially causing the cooling need to rise: it increases by a factor 2 for a silica glass. Heating demand is not as strongly impacted, because it also benefits from the urban environment: solar heating can be highly non-uniform between façades with different orientations, leading to significant heat transfer between adjacent buildings. These variations in energy demand are enough to change which passive window is optimal, the emissive radiative cooler being more efficient in the case (while IR-reflecting windows were more efficient for the urban environment).

The heating and cooling needs of a building strongly depend on its location, due to the high variability of key quantities such as the mean temperature or the solar irradiation (see Fig. S4). Therefore, the optimal passive or switchable window will be different from one location to another. To illustrate this, we show in Fig. S5 the electrical load of a building located in Stockholm, where the mean temperature is almost 10 °C lower and the yearly global horizontal irradiance 40% lower than in Barcelona. For simplicity, we considered the same atmospheric properties as in Barcelona (see Table S1). Because of the cold climate of Stockholm, the solar heating window becomes the best of the four passive windows in urban conditions, while the IR-reflecting one is optimal in rural conditions. In addition, a building equipped with a reflecting window already exhibit almost no cooling need in summer: in such a climate, it should be sufficient to switch the properties in either the NIR or the ATW (instead of both in Barcelona) to achieve almost ideal performance, simplifying the design of the smart window.

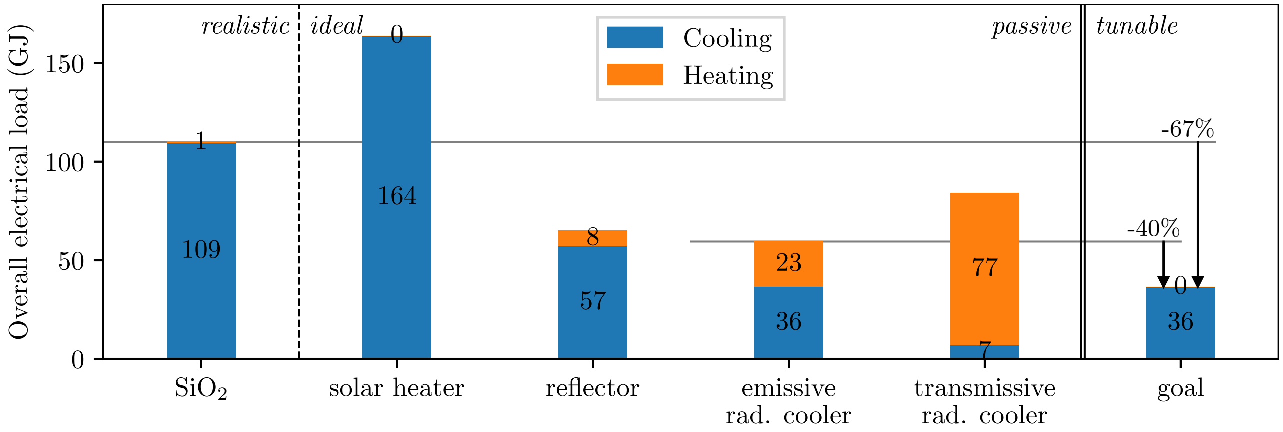

Fig. S3: Yearly electrical load of a building in a rural environment at the latitude of Barcelona due to heating and cooling, considering a building with dimensions $L = 20$ m and $H = 10$ m.

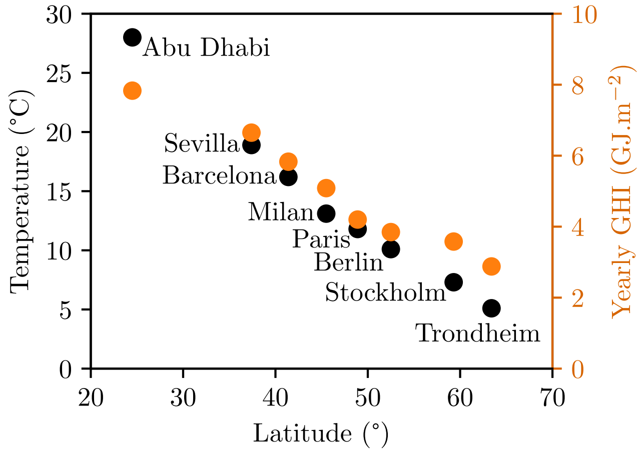

Fig. S4: Influence of the location latitude on the average temperature and global horizontal irradiance (GHI) from the Sun over the year.

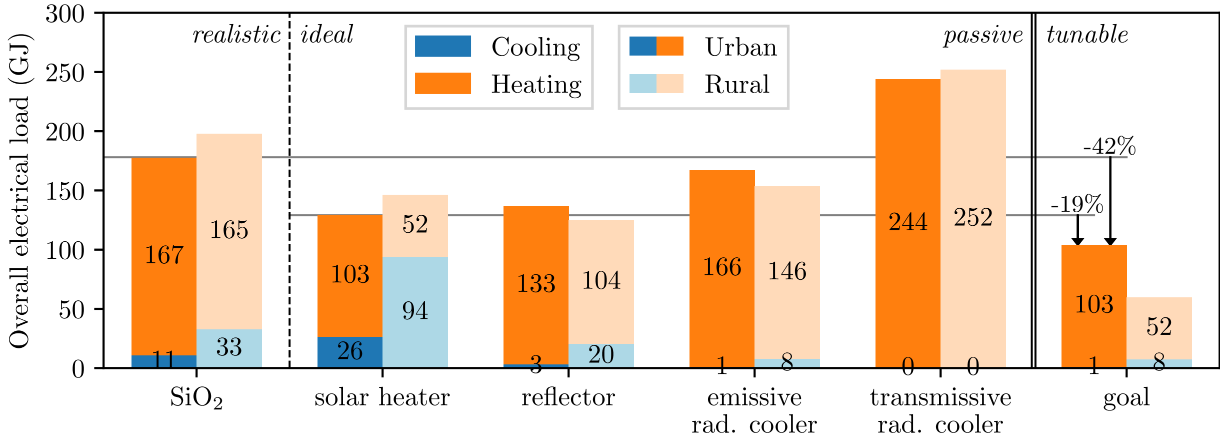

Fig. S5: Yearly electrical load of a building in Stockholm due to heating and cooling, considering a building with dimensions $L = 20$ m and $H = 10$ m. The results are given for both urban (5 m distance between buildings) and rural environments.

S3 Broadband vs. selective radiative cooling

In the main article, only selective radiative cooling has been considered, with the radiative cooling window emitting exclusively in the ATW. It is also possible to design a broadband radiative cooling structure emitting in the whole MIR instead. Such a structure not only cools down by sending radiation to space through the atmospheric window, but also by exchanging heat with its environment. Of course, the latter phenomenon can only cool the structure if the environment is colder: broadband coolers therefore reach large cooling power only when at sufficiently high temperatures, and selective coolers are necessary to attain low temperature [11]. In the case of windows, the radiative cooling structure not only faces the cold atmosphere, but also the ground and other buildings which are at a temperature similar to the window. This mitigates the interest of broadband cooling, the additional cooling achieved by exchanging radiation with the atmosphere being balanced with the heat transfer with the buildings and the ground. This is illustrated in Fig. S6, in which the cooling demand of a building appears to be comparable when equipped with broadband or selective cooling windows. Since broadband cooling windows exhibit worse performance in heating mode, we focused on selective windows in the main article. Still, note that switchable window operating as broadband coolers in summer could also be performant, and the choice between broadband and selective cooling for switchable windows should eventually be made based on the performance achievable by realistic structures, and on the simplicity, the economic and the environmental costs of such structures.

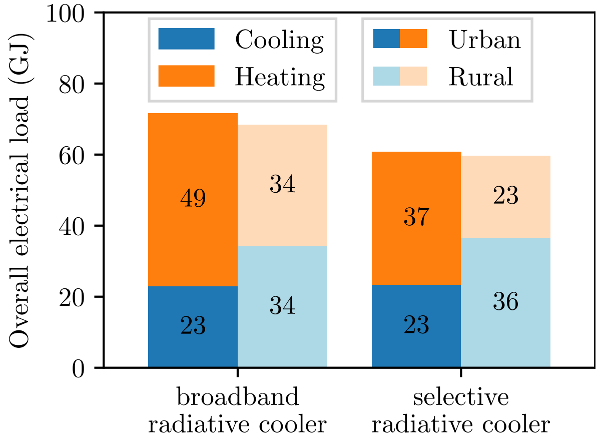

Fig. S6: Yearly energy demand of buildings in Barcelona equipped with broadband (i.e., in the whole MIR) or selective (i.e., in the ATW only) emissive radiative cooling windows.

S4 Details on the resonant behavior in the atmospheric window

Although the cross-shaped resonators form a periodic structure on the top surface of the window, note that the resonant behavior does not come from this periodicity. The resonators should rather be understood as two perpendicular plasmonic antennas, which resonate with radiation with polarization parallel to their main dimension due to surface plasmon polaritons [12]. In Fig. S7, we represent the local electric field and absorption below the resonator (i.e. at $x = 0$ or $y = 0$) considering an incident electric field with unit amplitude in the x direction. When the resonator is in its metallic phase (in summer), we can observe a strong enhancement of the x component of the field close to the edges of the resonator (panel (d)), which is a classical result for resonant antennas [12]. As expected, this enhancement cannot be observed for the orthogonal antenna, which is no longer parallel to the polarization (panel (e)). This plasmonic resonance causes the resonator to strongly absorb - thus emit - at certain wavelengths (panel (f)). Coherently with the literature [12], the resonant wavelength can be tuned by controlling the length and the section of the antenna, and exhibit a linear variation with the antenna length which here is approximately $\lambda_{\text{res}} = 2.4L + 0.5$.

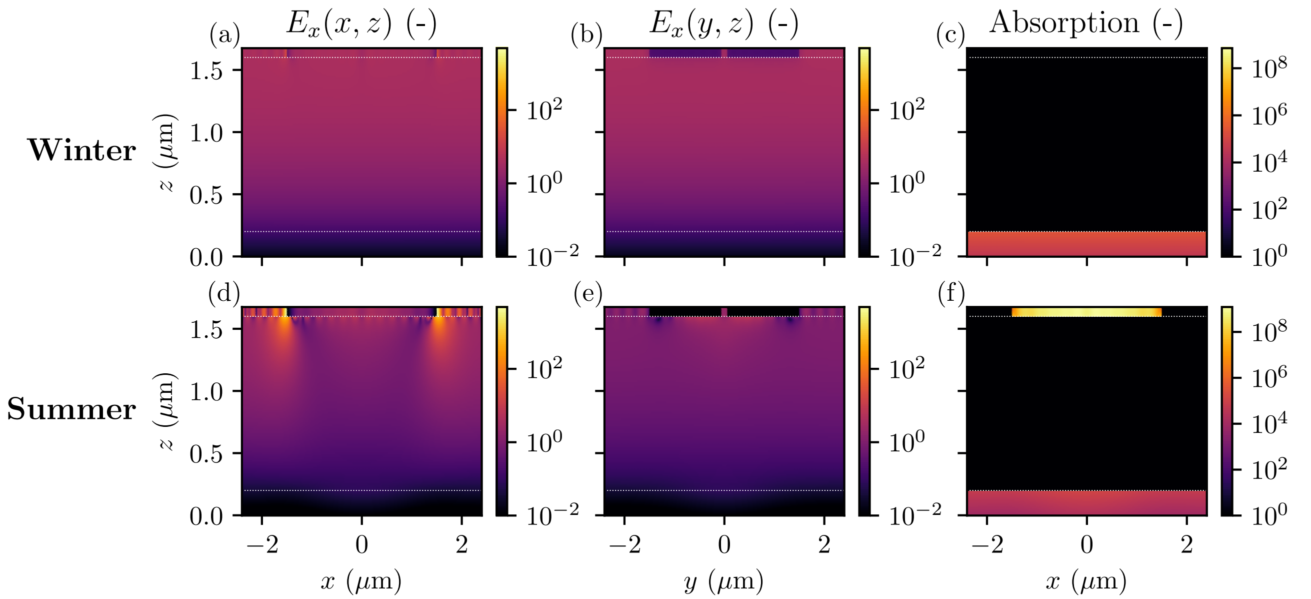

Fig. S7: (a-b), (d-e) x-component of the electric field in the structure at a wavelength $\lambda = 10$ μm, respectively in winter and summer operation. (c,f) Local absorption for $\lambda = 10$ μm in both seasons, in the (x-z) plane. In all figures, dashed lines represent the boundary between layers. All the quantities drawn are unitless, the electric field incident on the structure being assumed to have unit amplitude and to be along the x-direction.

S5 Impact of metallic losses on tunable window performance

In Fig. S8 are represented the variation of the mean visible transparency and the ATW emissivity modulation of the MIR-tunable structure shown in Fig. 5 of the main text, as a function of the damping coefficient γ of the transparent conductive material (TCO) and of the phase-change material (PCM) in metallic phase. This parameter is directly related to metallic losses, i.e. to the absorptivity of the material. Regarding the PCM, γ has almost no influence on the window transparency, its resonant behavior being limited to the MIR range. Although the emissivity modulation strongly varies with the damping coefficient, this is not a concern for the smart window performance as the resonator geometry can be optimized to achieve large $\Delta\varepsilon$ for any γ. This is the reason why the value selected in the main article, indicated by a dot, is close to a maximum. On the contrary, the TCO should only reflect or transmit radiation, and should consequently have the lowest damping coefficient possible to minimize absorption and emission. From Fig. S8, it appears that γ_{TCO} should at least be lower than 0.1 eV, and ideally below 10 meV to limit the influence of absorption on the window performance. This is why $\gamma_{\mathrm{TCO}} = 10$ meV has been chosen in the main article.

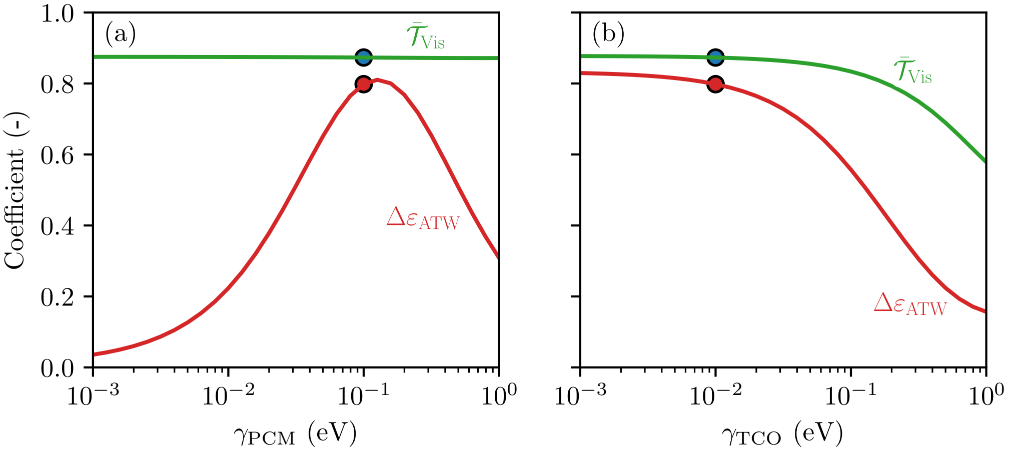

Fig. S8: Influence of the damping coefficient γ_{Drude} of (a) the PCM and (b) the TCO on the visible transmittance and ATW emissivity modulation of the MIR-tunable structure shown in Fig. 5 of the main text, at normal incidence. The dots indicate the values considered in the main article. While the resonator structure can be optimized to make the structure efficient for any γ_{PCM}, the TCO damping coefficient should be as low as possible to prevent parasitic absorption.

References

[1] Meteostat. https://meteostat.net/en/.
[2] Elies Campmany, Joan Bech, Javier Rodríguez-Marcos, Yolanda Sola, and Jerónimo Lorente. A comparison of total precipitable water measurements from radiosonde and sunphotometers. *Atmospheric Research*, 97:385–392, 2010.
[3] Eubrewnet. https://eubrewnet.aemet.es/eubrewnet.
[4] Aerosol Optical Depth at Barcelona. https://global-evaluation.atmosphere.copernicus.eu/aerosol/aod-aeronet-shortlist/europe-barcelona.
[5] Kevin S. Anderson, Clifford W. Hansen, William F. Holmgren, Adam R. Jensen, Mark A. Mikofski, and Anton Driesse. Pvlib python: 2023 project update. *Journal of Open Source Software*, 8(92):5994, 2023.
[6] Planetary Spectrum Generator. https://psg.gsfc.nasa.gov/.
[7] Yeonghoon Jin and Mikhail A. Kats. A gradient atmospheric model reveals enhanced radiative cooling potential and demonstrates the advantages of broadband emitters, 2024. *Preprint*, arXiv:2406.00572.
[8] Simone Kotthaus, Thomas E. L. Smith, Martin J. Wooster, and C. S. B. Grimmond. Derivation of an urban materials spectral library through emittance and reflectance spectroscopy. *ISPRS Journal of Photogrammetry and Remote Sensing*, 94:194–212, 2014.
[9] J. C. Chiu, J. A. Holmes, R. J. Hogan, and E. J. O'Connor. The interdependence of continental warm cloud properties derived from unexploited solar background signals in ground-based lidar measurements. *Atmospheric Chemistry and Physics*, 14:8389–8401, 2014.
[10] D. Serrano, M. J. Marín, M. Núñez, S. Gandía, M. P. Utrillas, and J. A. Martínez-Lozano. Relationship between the effective cloud optical depth and different atmospheric transmission factors. *Atmospheric Research*, 160:50–58, 2015.

[11] Shanhui Fan and Wei Li. Photonics and thermodynamics concepts in radiative cooling. *Nature Photonics*, 16:182–190, 2022.

[12] Frank Neubrech, Christian Huck, Ksenia Weber, Annemarie Pucci, and Harald Giessen. Surface-Enhanced Infrared Spectroscopy Using Resonant Nanoantennas. *Chemical Reviews*, 117:5110–5145, 2017.
